# Post-diagnosis physical activity in relation to mortality among gynecological cancer survivors

**DOI:** 10.1007/s10552-026-02211-7

**Published:** 2026-07-04

**Authors:** Alina Hamann, Pauline Benker, Michael F. Leitzmann, Michael J. Stein

**Affiliations:** 1 Department of Epidemiology and Preventive Medicine, University of Regensburg, Franz-Josef-Strauss-Allee 11, 93053 Regensburg, Germany; 2 Institute of Epidemiology, Helmholtz Zentrum München, German Research Center for Environmental Health (GmbH), Ingolstädter Landstraße 1, 85764 Neuherberg, Germany

**Keywords:** Post-diagnosis physical activity, Gynecological cancer, Mortality, Meta-analysis

## Abstract

**Purpose:**

Physical activity may play a supportive role in cancer survivorship. However, evidence on the association between post-diagnosis physical activity and mortality among women with gynecological cancer remains limited and inconsistent.

**Methods:**

We conducted a systematic review of the literature published between 1949 and January 2026. Eligible observational studies were identified, and random-effects meta-analyses were performed to estimate pooled hazard ratios (HRs) and 95% confidence intervals (CIs) for the association between post-diagnosis physical activity and all-cause mortality among women diagnosed with gynecological cancer.

**Results:**

A total of ten eligible studies on endometrial, ovarian, and cervical cancer were included, collectively reporting 3,867 deaths. High levels of post-diagnosis physical activity, compared with low levels, were associated with lower mortality (HR: 0.65; 95% CI 0.54–0.78). This inverse relationship was evident in both endometrial and ovarian cancer survivors (endometrial cancer: HR: 0.60; 95% CI 0.43–0.83; ovarian cancer: HR: 0.71; 95% CI 0.58–0.86). Medium levels of physical activity tended to be inversely associated with mortality (HR: 0.88; 95% CI 0.76–1.02).

**Conclusion:**

Higher levels of physical activity after a gynecological cancer diagnosis were associated with improved survival. The results suggest that physical activity may represent a modifiable lifestyle factor with the potential to improve long-term outcomes among gynecological cancer survivors.

**Implications for cancer survivors:**

This supports the potential value of integrating physical activity into survivorship care, although further high-quality prospective studies are needed to strengthen causal inference.

**Supplementary Information:**

The online version contains supplementary material available at 10.1007/s10552-026-02211-7.

## Introduction

In 2022, gynecological cancers, including endometrial, ovarian, and cervical cancer, accounted for almost 1.5 million new cancer cases and 680,000 deaths globally [1], with endometrial cancer being the fourth most common cancer and the sixth most lethal cancer in women [2]. Cancer survivors face recurrence risk as well as higher morbidity and mortality [3].

Modifiable lifestyle factors such as physical activity may positively influence cancer survivors’ prognosis [4]. Current World Health Organization (WHO) guidelines recommend 150–300 min per week of moderate-to-vigorous physical activity (MVPA) in order to reduce treatment-related side effects and improve overall survival [5]. Despite these recommendations, nearly half of gynecological cancer survivors report being insufficiently active, largely due to treatment-related side effects and disease-specific barriers [6].

Prior research has investigated survival benefits of physical activity among survivors of breast and colorectal cancer, two of the most common cancers globally [7, 8]. These findings may not be directly generalizable to all malignancies. Gynecological cancers differ in key biological and clinical characteristics, including hormonal milieu, tumor biology, and treatment modalities, which may plausibly modify the relationship between post-diagnosis physical activity and survival outcomes. Evidence regarding this association is also accumulating for individuals with gynecological cancers. A 2020 meta-analysis investigated post-diagnosis physical activity by different cancer sites and, based on four studies, reported a 37% reduced mortality risk among gynecological cancer survivors [9].

Despite the increasing interest in post-diagnosis physical activity, evidence regarding gynecological cancer survivors is still limited. To address this research gap, we conducted a systematic review of the existing evidence on the association between post-diagnosis physical activity and mortality among women with endometrial, ovarian, and cervical cancers. Such evidence may contribute to future guidelines, survivor care programs, or patient counseling.

## Methods

This systematic review and meta-analysis was conducted according to the Preferred Reporting Items for Systematic Reviews and Meta-Analyses (PRISMA) [10].

### Literature search strategy

In order to identify the entirety of relevant studies, we conducted a wide literature search in MEDLINE, Embase and CINAHL using the following search terms: (“physical activit*” OR exercise OR “motor activity”) AND (cancer OR neoplasm* OR carcinoma OR adenocarcinoma OR sarcoma OR tumor* OR malignanc* OR “abnormal cells”) AND (survivors OR survivor OR survivorship OR patients OR patient) AND (mortalit* OR fatal* OR “death rate” OR survival OR recurrence OR progression OR outcome* OR prognos*). Thus, we included relevant synonyms and keywords for physical activity, cancer, and mortality. We initially set no restrictions on cancer type to avoid missing relevant studies and subsequently narrowed the scope to gynecological cancers during full-text screening. There were no limitations set for geographical region or publication date, but the analyses were confined to articles studying human populations by using the respective filter in MEDLINE, CINAHL, and Embase*.* Additionally, the reference lists of all included studies were reviewed to identify further relevant literature. The primary search was carried out until 15 January 2026, and e-alert notifications were set in MEDLINE to capture all studies published after that date.

In this study, the term “cancer survivors” refers to individuals from the time of cancer diagnosis onwards, regardless of disease stage or treatment status.

### Eligibility screening

Two independent reviewers (AH, PB) assessed the eligibility of studies by screening titles and abstracts. Inclusion criteria for full-text reviews were physical activity measurement as exposure and mortality or survival parameters (recurrence, progression, etc.) as outcomes. If studies were found to have unclear relevance, we included them for full-text review. The full texts of the remaining studies considering sex-specific cancers or overall cancer (cancer of any type) were further reviewed by both reviewers to identify studies on gynecological cancers. Studies were kept for data extraction if the exposure was post-diagnosis physical activity, all-cause mortality was reported, and adequate risk estimates (hazard ratio (HR) or odds ratio with confidence intervals (CI)) were used. If conflicts arose between the decisions made by the reviewers, they were discussed in consultation with a third reviewer (MJS) and resolved by consensus.

### Data extraction

The following data were extracted and placed in a data collection chart: author and title, publication year, geographical location, cancer type, physical activity type and activity units, outcome type, study design, number of participants, number of deaths, and any potential sub-analysis conducted. Furthermore, we extracted the given HR and 95% CI (for high/medium/low physical activity), physical activity cut-offs, follow-up time, and adjustment factors. If the HR was only given for low compared to high activity levels, it was converted into the according reciprocal for comparability. We extracted information on leisure-time physical activity, which was expressed mostly as Metabolic Equivalent of Task-hours (MET-hrs) per week of MVPA. Otherwise, we converted exercise frequency and intensity of measured leisure-time activities into MET-hrs per week. MET-hrs represent the cumulative energy expenditure of physical activity, calculated as the product of the activity’s intensity expressed in METs and its duration in hours [11]. Data were extracted for a high physical activity category defined as the category with a cut-off closest to the WHO recommendation of more than 7.5 MET-hrs per week of MVPA. Where multiple higher activity categories were available, the estimate nearest to this threshold was selected. A medium category was extracted where reported. The low physical activity category was defined as the least active or no activity group in each study. All effect estimates were calculated relative to this lowest category, which served as the common reference group.

Post-diagnosis physical activity was defined as physical activity assessed after cancer diagnosis, measured at a single time point in each included study using self-reports.

### Study quality assessment

Quality and risk of bias of the included studies were assessed by a single reviewer (AH) using the Newcastle Ottawa Scale (NOS) [12]. The NOS assigns zero to nine points, where zero points indicate poor-quality studies, and nine points indicate high-quality studies. Three different domains were evaluated: a maximum of four points for selection, two points for comparability, and three points for outcome.

For selection bias, two points were awarded if the sample was representative of the exposed cohort, one point if physical activity was assessed through an interview as opposed to self-administered, and one point if the outcome of interest was absent at study onset.

For comparability bias, one point was awarded for adjusting for at least one cofounder and two points if the analysis was controlled for multiple confounders.

For outcome bias, one point was awarded if the outcome was determined through record linkage, one point if the study had a follow-up duration of more than three years, and one point if loss to follow-up was described, or if the study achieved complete follow-up.

### Statistical analysis

HRs and corresponding 95% CIs were extracted from each study. For meta-analysis, HRs were log-transformed to obtain effect size estimates. Each estimate was weighted by the inverse of its variance plus the between-study variance, such that studies with greater precision contributed more strongly, while accounting for between-study heterogeneity. The pooled effect was calculated as the weighted average of study-specific estimates. The uncertainty of the pooled effect was quantified using its standard error, derived from the sum of the study weights. Ninety-five percent CIs were obtained by adding and subtracting 1.96 times the standard error from the pooled estimate. Pooled estimates and their CIs were then exponentiated back from the logarithmic scale to the HR scale for interpretation. Each study contributed only once to a given meta-analysis; when results were available only by cancer-type subgroups, these subgroups were modeled independently to account for clinical heterogeneity and reduce within-study confounding. Subgroup analyses were conducted by cancer type (when at least two eligible studies were available) and by physical activity dose (exceeding WHO guidelines vs. below guidelines). Forest plots were generated to visualize study-specific and pooled estimates [13].

We conducted all statistical analyses using R (version 4.5.0) and the metafor package [14].

The protocol was registered in PROSPERO under the registration number CRD420251268969.

## Results

### Systematic review

**Fig. 1:**
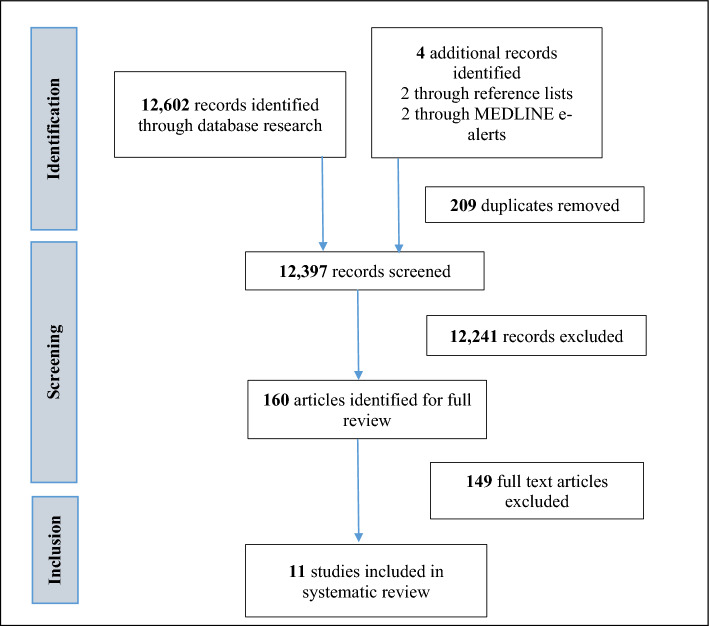
Flow diagram showing the literature search and study selection process for the systematic review and meta-analysis

Our literature search in MEDLINE, Embase, and Cinahl provided a total of 12,602 articles, two additional articles from the reference lists of included studies, and two by MEDLINE e-alerts. After removal of duplicates, 12,397 records remained for title and abstract screening, of which 160 were eligible for full-text review. Of these, 149 studies were excluded due to either reporting an inappropriate physical activity exposure (e.g., pre-diagnosis physical activity, short-term exercise intervention trials) or not reporting all-cause mortality as an outcome. This left 11 cohort studies for final inclusion in our systematic review (Table 1, Fig. 1). Among these studies, six assessed post-diagnosis physical activity in women with endometrial cancer and together accounted for 2,342 deaths, five examined women with ovarian cancer with a total of 1,444 deaths, one focused on women with cervical cancer, reporting 30 deaths, and one included women with female genital cancer, reporting 51 deaths. The two studies that assessed both endometrial and ovarian cancer separately were treated as individual studies in our analysis.

**Table 1:** Characteristics of the studies included in the systematic review

Author, year, reference	Cancer type	Title	Physical activity	Number of patients	Number of deaths	Quality score (NOS)
Rees-Punia, 2025 [23]	Endometrial, Ovarian	Leisure-time physical activity after diagnosis and survival by cancer type: a pooled analysis	MVPA: 0 to < 7.5, 7.5 to < 15, ≥ 15 MET-hr/wk	3,686 (endometrial), 1,334 (ovarian)	1,421 (endometrial), 783 (ovarian)	9
Lavery, 2024 [15]	Endometrial, Ovarian	Pan-Cancer Analysis of Post-Diagnosis Exercise and Mortality	Sessions/week: ≥ 4 × 30 min moderate/wk (= 6–12 MET-hr/week) or ≥ 2 × 20 min strenuous/wk, any activity below guideline (incl. 0/wk)	361 (endometrial), 109 (ovarian)	95 (endometrial), 56 (ovarian)	9
Arem, 2016 [16]	Endometrial	Body mass index, physical activity, and television time in relation to mortality risk among endometrial cancer survivors in the NIH-AARP Diet and Health Study cohort	MVPA: 0, 0.1– < 15, 15 + MET-hr/week	580	91	8
Friedenreich, 2020 [17]	Endometrial	Prospective Cohort Study of Pre- and Post-diagnosis Physical Activity and Endometrial Cancer Survival	Tertiles of domain-specific MET-hr/week	425	60	9
Tarasenko, 2018 [19]	Endometrial	Muscle-strengthening and aerobic activities and mortality among 3 + year cancer survivors in the U.S	Exercise frequency: ≤ 1 session/wk of 10 min, ≥ 1 session/wk for 10–150 min, ≥ 150 min/wk of moderate PA or 75 min/wk of vigorous PA	1,038	207	9
Bates-Fraser, 2025 [18]	Endometrial	Every move matters: physical activity, walking, sedentary behavior, and endometrial cancer survival	MVPA: 0 MET h-/wk, > 0 to < 7.5 MET-hr/wk, 7.5 to < 15 MET-hr/wk, 15 to < 22.5 MET-hr/wk, 22.5–30 MET-hr/wk	946	468	8
Abbott, 2018 [20]	Ovarian	Recreational physical activity and survival in African-American women with ovarian cancer	Total PA: 0, > 0–9, and > 9 MET-hrs/week	264	80	9
Hansen, 2020 [21]	Ovarian	A healthy lifestyle and survival among women with ovarian cancer	Tertiles of Leisure time PA	512	122	8
Wang, 2021 [22]	Ovarian	Pre-diagnosis and post-diagnosis leisure time physical activity and survival following diagnosis with ovarian cancer	Leisure time PA in MET-hr/wk (< 1.5, 1.5–7.5, > 7.5)	669	403	9
Kim, 2016 [24]	Cervical	Health-Related Quality of Life and Sociodemographic Characteristics as Prognostic Indicators of Long-term Survival in Disease-Free Cervical Cancer Survivors	Exercise frequency: > 30 min on > 5 days per week, anything below	860	30	8
Jiang, 2025 [25]	Female Genital	Association between accelerometer-measured physical activity and mortality in cancer survivors: A prospective cohort study from UK Biobank	Exercise frequency: > 272 min MVPA per week	505	51	9

*PA* physical activity, *No* number, *hr* hours, *wk* week, *MET* metabolic equivalent of task

Quality assessment revealed that seven studies achieved the maximum score of 9 points. Three studies scored 8 points due to the use of self-administered physical activity questionnaires, and one study scored 8 points because it included exclusively long-term disease-free cancer survivors, resulting in a non-representative population. Most studies adjusted for important covariates such as age, cancer treatment or stage, body mass index, comorbidities or health status, and smoking status. Covariates were largely self-reported, although six studies used medical records to verify cancer treatment or stage. However, the number, type, and quality of covariates varied across studies. For example, only three studies assessed alcohol consumption and only two accounted for female-specific factors such as hormone replacement therapy (Supplementary Table 1).

Overall, four of the six included studies on endometrial cancer reported a significant survival benefit, whereas two of the five studies on ovarian cancer showed a significant benefit [15–23]. Among these studies, the largest was a pooled analysis of post-diagnosis physical activityKlicken oder tippen Sie hier, um Text einzugeben. including 3,686 endometrial cancer survivors and 1,334 ovarian cancer survivors [23]. The results from that study showed a significant survival benefit for endometrial cancer survivors engaging in at least the recommended levels of leisure-time physical activity, whereas the association among ovarian cancer survivors was directionally similar but not statistically significant.

One study, with a particularly long follow-up of 30 years, reported significantly lower mortality among ovarian cancer survivors [22]. The authors observed a 29% lower mortality for individuals engaging in at least recommended levels of physical activity during the first 4 years after diagnosis. This study also stratified by tumor stage, where the association was more pronounced among survivors with stage I/II tumors compared to those with stage III/IV tumors. Regarding cervical cancer, we only included one study that found a significantly lower mortality among women who engaged in regular physical activity [24].

In one study assessing the composite of female genital cancers, the authors found no statistically significant association between post-diagnosis physical activity and mortality [25]. Since this study used a composite outcome, it was not included in the meta-analysis.

### Meta-analysis

**Fig. 2:**
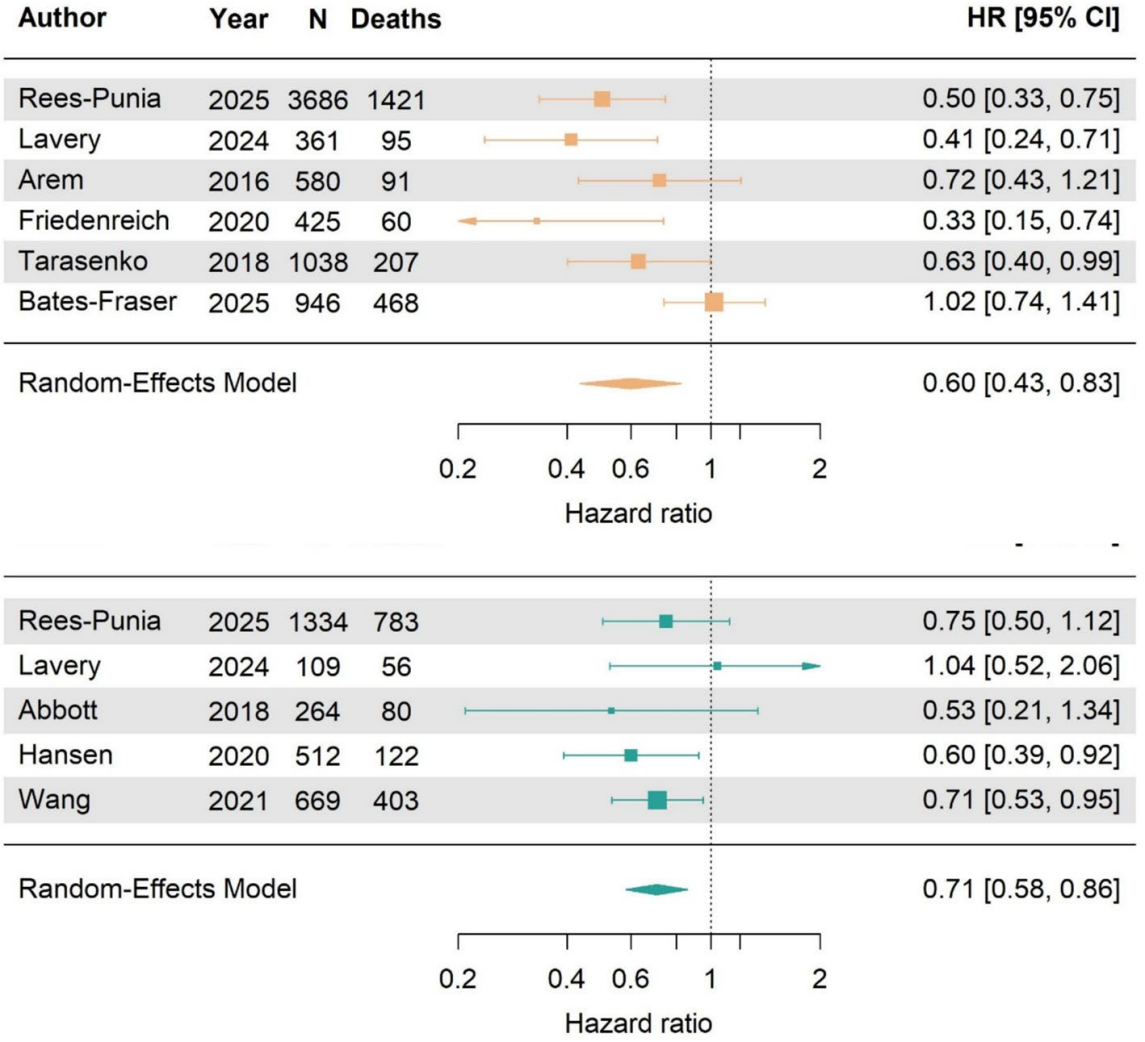
Hazard ratios for all-cause mortality among survivors of gynecological cancers comparing high physical activity (exceeding guidelines of 7,5 MET-hours per week) and the lowest physical activity category in studies of endometrial cancer (top) and ovarian cancer (bottom)

Among the included studies, four studies investigated endometrial cancer survivors and two investigated ovarian cancer survivors (Fig. 2). Subgroup analyses indicated an inverse association between high post-diagnosis physical activity and mortality (compared with the reference group of no or low physical activity) among both endometrial cancer survivors (HR: 0.60; 95% CI 0.43–0.83) and ovarian cancer survivors (HR: 0.71; 95% CI 0.58–0.86). Meta-analysis of all cancers combined yielded a pooled HR of 0.65 (95% CI 0.54–0.78), for high versus low levels of physical activity (Supplementary Fig. 1).

### Secondary analysis

**Fig. 3:**
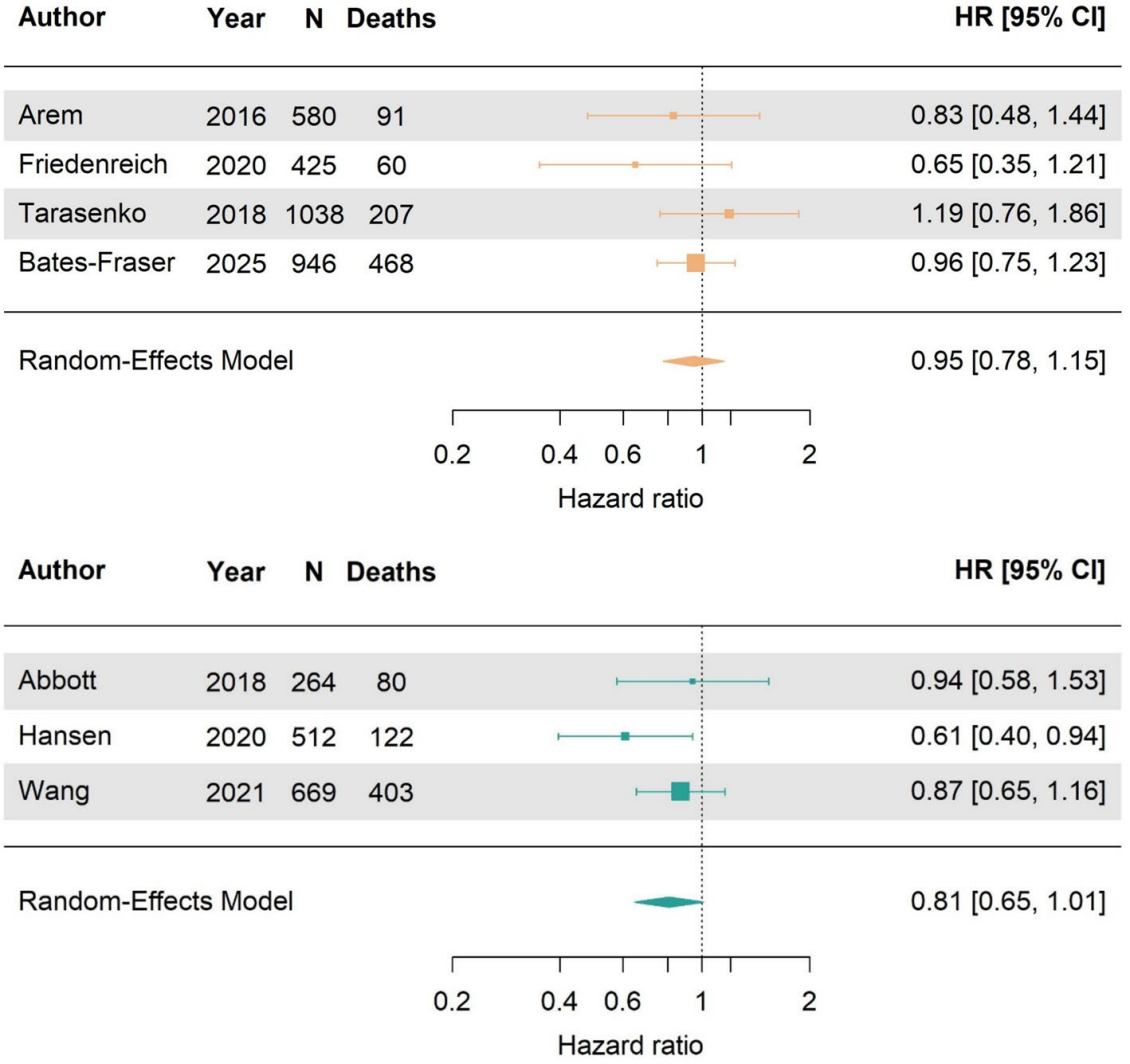
Hazard ratios for all-cause mortality among survivors of gynecological cancer comparing medium physical activity (below the WHO recommendation of 7.5 MET-hrs per week) with the lowest physical activity category in studies of endometrial cancer (top) and ovarian cancer (bottom)

Regarding medium physical activity levels (below WHO recommendations) compared with low or no physical activity, the pooled estimate for gynecological cancer survivors was 0.88 (95% CI 0.76–1.02) (Supplementary Fig. 2). Subgroup analyses showed an attenuated HR of 0.95 (95% CI 0.78–1.15) for endometrial cancer survivors and an inverse tendency with a HR of 0.81 (95% CI 0.65–1.01) for ovarian cancer survivors (Fig. 3).

Visual inspection of the funnel plot suggested a left-sided asymmetry (Supplementary Fig. 3). However, both Begg’s rank correlation test (*p* = 0.31) and Egger’s regression asymmetry test (*p* = 0.08) indicated no statistically significant publication bias. This discrepancy is likely due to the small number of included studies and the consistent direction of effect sizes.

## Discussion

Our meta-analysis of ten individual studies demonstrated a statistically significant reduction in mortality among women with gynecological cancer engaging in recommended physical activity after cancer diagnosis. Medium levels of post-diagnosis physical activity only tended to be inversely associated with mortality. The summarized evidence indicates that engaging in higher levels of physical activity after a diagnosis of gynecological cancer is associated with substantial reductions in mortality.

Meta-analyses of well-studied cancers, such as breast and colorectal cancers, have consistently shown that physical activity is associated with reduced mortality, with dose–response analyses indicating greater survival benefits at higher levels of activity [26, 27]. In contrast, research on gynecological cancers has largely focused on the preventive relation of pre-diagnosis physical activity [28, 29], with limited data on post-diagnosis activity, which is critical for informing survivorship care. In this context, our work expands the existing literature on survival benefits of post-diagnosis physical activity to include gynecological cancer types.

One prior meta-analysis examined post-diagnosis physical activity and its association with mortality among women with gynecological cancers. In line with our study, the authors reported a lower mortality in this population; however, their study was based on only four studies and did not examine individual gynecological cancer types [9]. In addition, we examined high levels as well as medium levels of post-diagnosis physical activity compared to no physical activity or low levels. Notably, the HR for high physical activity versus low physical activity was lower for endometrial than ovarian cancer survivors (HR: 0.60 vs. 0.71), whereas for medium versus low activity, the HR was slightly lower for ovarian than endometrial survivors (HR: 0.81 vs. 0.95). However, these differences may be driven by a limited number of studies within subgroups or differences in exposure classification across studies and should therefore be interpreted cautiously.

Multiple pathophysiological mechanisms may underlie our observed associations. Among gynecological cancer survivors, regular physical activity may reduce critical risk factors such as obesity, insulin resistance, and estrogen exposure, ultimately improving overall prognosis and decreasing cancer-specific mortality [30, 31]. In particular, regular physical activity enhances insulin sensitivity and decreases circulating insulin and insulin-like growth factor 1 concentrations, thereby attenuating proliferative, angiogenic, and anti-apoptotic signaling pathways that contribute to tumor progression [32]. Moreover, exercise has been shown to lower systemic levels of C-reactive protein and proinflammatory cytokines [33], leading to a reduction in chronic inflammation, which plays a central role in tumorigenesis. Physical activity also exerts immunomodulatory effects by promoting an immunoprotective milieu, characterized by increased activity and number of natural killer cells and cytotoxic T lymphocytes [32, 33].

Several studies have investigated the relation between physical activity and mortality among cancer survivors, indicating significant benefits, including gynecological cancer survivors [15–22, 24]. However, many survivors, particularly those with ovarian cancer [34], do not achieve the recommended levels of physical activity. Contributing factors include treatment-related side effects associated with aggressive therapies, or fear of injury during treatment [35]. Additional determinants of physical activity among cancer survivors may include stigma, healthcare access, social support, health literacy, psychosocial factors, and financial constraints. Nonetheless, our findings support the importance of encouraging cancer survivors to engage in physical activity, as this represents a non-pharmacological approach to improving cancer outcomes.

Future research should include randomized controlled trials investigating long-term exercise interventions in cancer survivors. In addition, future studies may benefit from incorporating objective measures of physical activity, as these can complement self-reported assessments and reduce potential measurement bias associated with questionnaire-based approaches. Furthermore, longitudinal analyses stratified by treatment type or tumor stage may provide more detailed insights into associations across survivor subgroups.

### Strength and limitations

A strength of our study is the wide literature search, ensuring the inclusion of all relevant articles on this topic. In total, we analyzed studies reporting 3,867 deaths among 11,289 cancer survivors. An additional advantage is our use of random-effects models, which account for variability between studies and provide more generalizable results. Furthermore, we conducted subgroup analyses by cancer type and activity dose, allowing us to explore potential differences in associations across specific populations. Findings were consistent across studies employing different methodologies, supporting the validity of our conclusions.

Limitations of our study include the common reliance on self-reported assessment of physical activity. Since self-reported physical activity is generally subject to non-differential misclassification, this may have attenuated the observed associations and resulted in an underestimation of the true effects. The included studies assessed physical activity at a single post-diagnosis time point. Consequently, changes in physical activity over time could not be captured. Moreover, definitions of medium or high levels of physical activity varied across studies. Therefore, we used activity categories most closely aligned with WHO recommendations to facilitate public health communication. Our meta-analysis did not assess different physical activity domains, such as occupational activity, light-intensity movement, or strength training, which may also contribute to mortality outcomes in cancer survivors. Potential residual confounding due to factors such as dietary habits, body mass index, tumor stage, or socioeconomic status cannot be ruled out. However, we only extracted fully adjusted risk estimates to minimize such potential bias. For cervical cancer, the number of available studies remains limited as only one study was included. Finally, given the observational nature of the included studies, causal inference remains uncertain.

## Conclusion

Our study demonstrates that higher levels of post-diagnosis physical activity are associated with improved overall survival among women living with gynecological cancer. Given its accessibility, safety, and broad health benefits, physical activity should be considered a key component of gynecological cancer care.

## Supplementary Information

Below is the link to the electronic supplementary material.

Supplementary file1 (DOCX 569 KB)

## Data Availability

No datasets were generated during the current study.
