# Supplementary material for: Post-diagnosis physical activity in relation to mortality among gynecological cancer survivors
Source: Cancer Causes Control. 2026 Jul 4;37(8):121. doi: 10.1007/s10552-026-02211-7 (PMC13332953; doi:10.1007/s10552-026-02211-7)
Supplement: Supplementary file 1 — Supplementary file1 (DOCX 569 KB) [file 10552_2026_2211_MOESM1_ESM.docx]

**Post-diagnosis Physical Activity in Relation to Mortality among Gynecological Cancer Survivors**

**Supplementary material**

**Supplementary Figure 1.** Hazard ratios for all-cause mortality among survivors of gynecological cancers, comparing high physical activity (exceeding guidelines of 7,5 MET-hours per week) with the lowest physical activity category

**Supplementary Figure 2.** Hazard ratios for all-cause mortality among survivors of gynecological cancers, comparing medium physical activity (below the WHO recommendation of 7.5 MET-hours per week) with the lowest physical activity category

**Supplementary Figure 3**. Funnel plot of included studies to evaluate publication bias

**Supplementary Table 1**. Covariate details of all studies included in the systematic review

**Corresponding author:** Michael J. Stein, Tel.: +49 941 944 521 6, Mail: michael.stein@helmholtz-munich.de, Department of Epidemiology and Preventive Medicine, University of Regensburg, Regensburg, Germany

**Supplementary Figure 1.** Hazard ratios for all-cause mortality among survivors of gynecological cancers, comparing high physical activity (exceeding guidelines of 7,5 MET-hours per week) with the lowest physical activity category

**
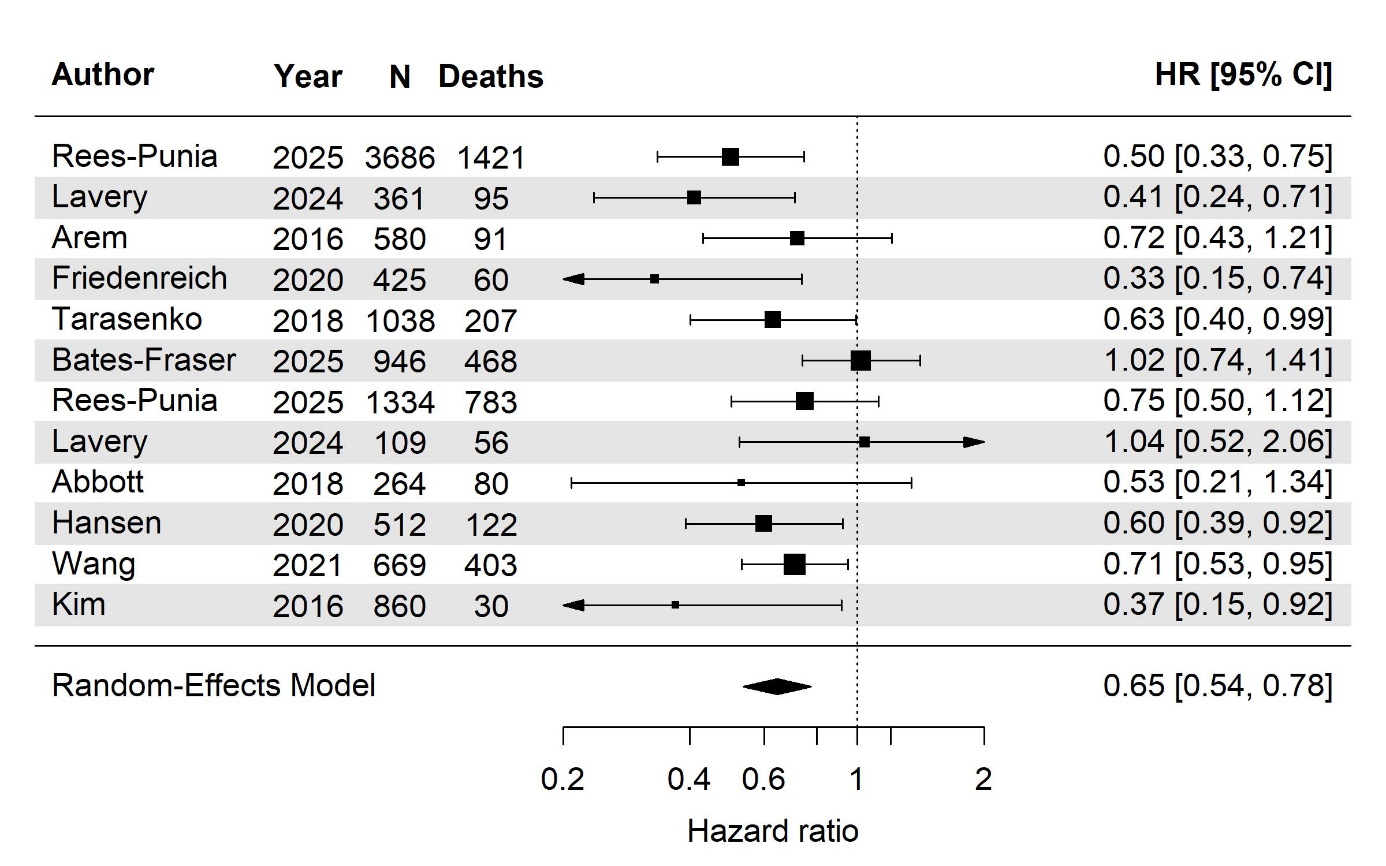
**

**Supplementary Figure 2.** Hazard ratios for all-cause mortality among survivors of gynecological cancers, comparing medium physical activity (below the WHO recommendation of 7.5 MET-hours per week) with the lowest physical activity category


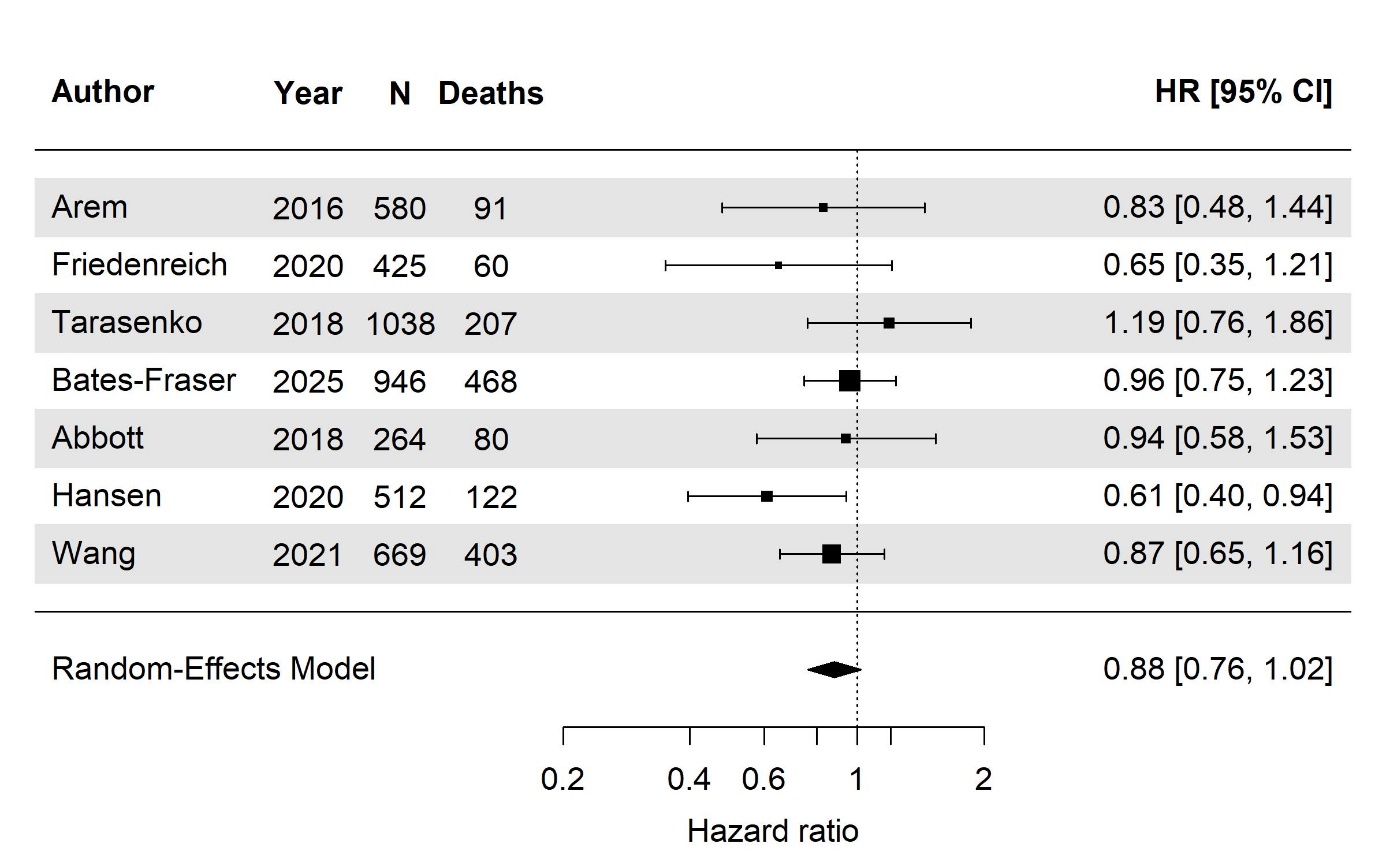


**Supplementary Figure 3**. Funnel plot of included studies to evaluate publication bias


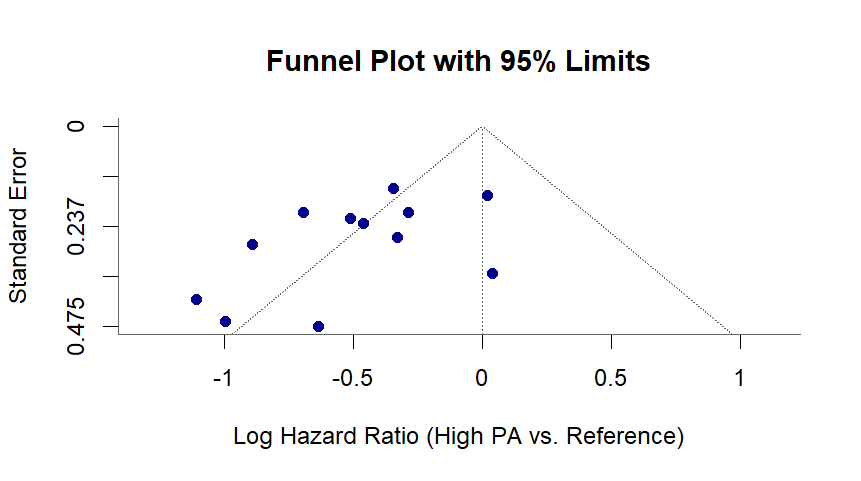


**Supplementary Table 1**. Covariate details of all studies included in the systematic review

| **Study, Year** | **Covariates** | **Assessment Methods** |
| --- | --- | --- |
| Rees-Punia, 2025 | age, sex, race/ethnicity, smoking status, alcohol use, cancer treatment and stage, BMI | self-reported, cancer registries, medical record verification |
| Lavery, 2024 | age, time since diagnosis, sex, race/ethnicity, BMI, comorbidities, smoking status, cancer treatment and stage | self-reported, medical record verification, follow-up assessments |
| Arem, 2016 | diagnosis age, grade, diabetes, age at menarche, hormone use, TV, BMI, health status, cancer treatment and stage | self-reported, follow-up assessments |
| Friedenreich, 2020 | age, grade, BMI, hormone use, menopausal status, family history, comorbidities, recurrence, pre-diagnosis PA, cancer treatment and stage | self-reported, follow-up assessments, anthropometric measurements |
| Tarasenko, 2018 | age, sex, race/ethnicity, education, marital status, insurance, activity limitations, smoking status, BMI, comorbidities, diagnosis age, health status | self-reported |
| Bates-Fraser, 2025 | age, education, marital status, race/ethnicity, pregnancy history, smoking status, alcohol use, treatment and stage, pre-diagnosis BMI | self-reported, cancer registries, medical records |
| Abbott, 2018 | age, cancer stage, region, comorbidities, education, income, pre-diagnosis physical activity | self-reported, follow-up survey |
| Hansen, 2020 | age, education, comorbidities, cancer stage, histology, residual disease | self-reported, medical records, follow-up assessments |
| Wang, 2021 | age, diagnosis year, histology, cancer stage, BMI, smoking status, NSAID | self-reported, follow-up assessments |
| Kim, 2016 | age, diagnosis age, cancer treatment and stage, time since diagnosis, follow-up time | self-reported, cancer registries, medical records |
| Jiang, 2025 | age, sex, Townsend deprivation score, ethnicity, smoking status, alcohol intake frequency, BMI, waist circumference, self-reported general health, cancer duration, cardiovascular disease history, diabetes history, long-standing illness, disability or infirmity, grip strength, FEV1, and wear season | self-reported, anthropometric measurements, medical records, spirometry, accelerometer data |
